# Public health round-up

**DOI:** 10.2471/BLT.24.010624

**Published:** 2024-06-01

**Aid to Rafah cut off**

Children in the city of Rafah in the occupied Palestinian territory where urgently needed aid was cut off on 7 May. On 8 May, the World Health Organization called for the removal of all obstacles to the delivery of humanitarian assistance into and across Gaza, at the scale required to respond to the humanitarian crisis.

**Figure:**
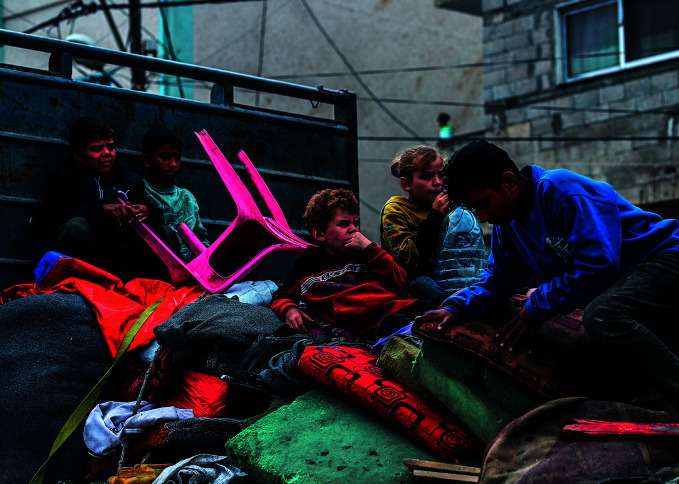
WHO

## Rafah crisis continues

The Israeli military seized control of the Gaza side of the Rafah and Kerem Shalom border crossings on 7 May, cutting the flow of humanitarian aid and adding to concerns about the already dire situation in the territory.

In an 8 May media briefing, World Health Organization (WHO) Director-General Tedros Adhanom Ghebreyesus called for the removal of all obstacles to the delivery of urgent humanitarian assistance into and across Gaza.

As of 8 May, an estimated 30–40 000 people had left Rafah for Khan Younis and Deir al-Balah, leaving more than 1.4 million people at risk in Rafah, including 600 000 children.

According to the Director-General, WHO had pre-positioned supplies in warehouses and hospitals, but without more aid, would not be able to support hospitals. He also noted that one of Rafah’s three hospitals – the An-Najjar hospital – had already shut down.

“WHO has no intention of withdrawing from Rafah and will stay and deliver alongside our partners,” the Director-General said.

WHO is currently coordinating the work of 20 Emergency Medical Teams in Gaza, comprised of 179 nationals from 30 countries, working alongside 800 local staff. The teams are embedded in 10 existing hospitals and have established five field hospitals.

https://bit.ly/3V0v2uy

## New dengue vaccine prequalified

WHO prequalified a new dengue vaccine. Developed by Takeda, a pharmaceutical company, TAK-003 is a live-attenuated vaccine containing weakened versions of the four dengue virus serotypes. It is the second dengue vaccine to receive WHO prequalification. 

In a 15 May statement, Dr Rogerio Gaspar, WHO director for regulation and prequalification, highlighted the significance of the step for global access and urged more vaccine developers to seek WHO prequalification.

https://bit.ly/4biCC9s

## Poliovirus in Malawi and Mozambique

An independent Polio Outbreak Response Assessment (OBRA) team recommended the closure of the wild poliovirus type 1 (WPV1) outbreak in Malawi and Mozambique, marking a significant milestone in the fight against polio in the African Region.

According to a 14 May WHO media release, the last WPV1 case in the African Region, linked to a strain circulating in Pakistan, was reported in Mozambique's Tete Province in August 2022. A total of nine cases were detected in Mozambique and neighbouring Malawi, where the outbreak was declared in February 2022.

In a coordinated response, more than 50 million children have been vaccinated to date against the virus in five countries in southern Africa.

The evaluation carried out by the OBRA team included two in-depth field reviews and supplementary data review, concluding that there is no evidence of ongoing wild poliovirus transmission.

https://bit.ly/44J0roq

## WHO progress report

The *World Health Organization 2023 results report* was published ahead of the Seventy-seventh World Health Assembly (WHA77), which ran from 27 May to 1 June 2024.

The report reflects on the progress made towards WHO’s ambitious “triple billion” targets set under the 13th General Programme of Work (GPW13) to spur efforts towards achieving – by 2025 relative to 2018 baselines – a one billion increase in the number of people benefiting from universal health coverage; another billion being protected from health emergencies; and another billion enjoying better health.

According to the report, as of 2023, 1.2 billion more people were enjoying better health relative to baseline; 429 million more people were benefiting from universal health coverage; and 599 million more people were better protected from health emergencies.

The report also presents progress toward the health-related sustainable development goals (SDGs), noting that, based on current trends, progress will be insufficient to reach the health-related targets set, with the exception of target 3a. on tobacco use.

https://bit.ly/3UTLMU1

## Pandemic agreements

State Parties to the International Health Regulations (2005) made progress on amendments to be put forward to WHA77.

According to a 26 April statement issued by WHO, the eighth meeting of the working group on amendments to the International Health Regulations saw significant progress towards finalizing a package of amendments.

WHO Director-General, Tedros Adhanom Ghebreyesus, underlined the historic nature of the opportunity to bolster global health security, emphasizing the need for equity and solidarity in the process.

The meeting coincided with the ninth meeting of the Intergovernmental Negotiating Body for a WHO instrument on pandemic prevention, preparedness and response. 

https://bit.ly/4bAkUhr

https://bit.ly/44EL5Bb

## Tracking avian influenza 

An assessment of the public health risk posed by avian influenza A(H5N1) viruses was published by WHO, the Food and Agriculture Organization (FAO), and the World Organisation for Animal Health (WOAH). The report considers the likelihood of transmission between and within different species.

Published on 23 April, the assessment reports that the human health risk posed by A(H5N1) to the general population is low, and low-to-moderate for people with exposure to infected birds or animals or contaminated environments.

However, because these viruses are constantly evolving and spreading, continuous monitoring is required and FAO, WHO and WOAH will continue to conduct and publish risk assessments for them.

https://bit.ly/3K1msp4

## HIV and syphilis transmission 

Belize, Jamaica, and St. Vincent and the Grenadines received WHO certification for eliminating the mother-to-child transmission of human immunodeficiency virus (HIV) and syphilis. The milestone was marked at an event organized by the Pan American Health Organization (PAHO) in Kingston, Jamaica, on 7 May with support from UN partners and with the participation of health ministers from the three countries.

To meet elimination targets, the countries focused on strengthening prevention and treatment services within primary health care and in maternal and child health, updating guidelines, ensuring the effective screening of pregnant women, monitoring of cases, and follow-up of infants exposed to HIV and syphilis.

“This achievement is a testament to years of dedication, hard work, and collaboration among governments, health professionals and communities”, said PAHO Director, Dr Jarbas Barbosa.

https://bit.ly/3wotmS5

**Cover photo**

A young woman receives an eye examination at the Khmer-Soviet Friendship Hospital, Phnom Penh, Cambodia.

**Figure:**
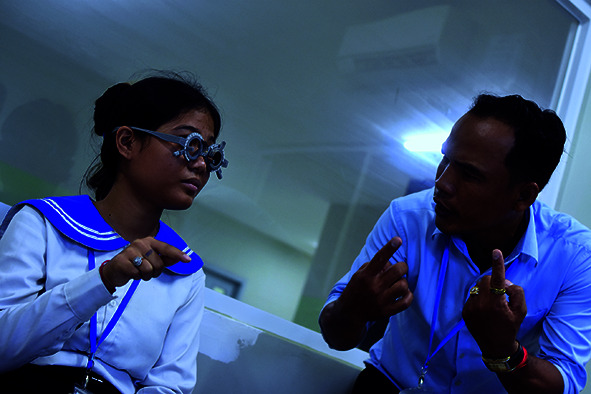
WHO/Miguel Jeronimo

## New intravenous catheter guidance

WHO issued the first global guidelines to prevent the occurrence of bloodstream and other infections caused by suboptimal intravenous catheter use during medical procedures.

Published on 9 May, the guidelines include recommendations for education, hand hygiene, intravenous catheter insertion and maintenance, as well as intravenous catheter selection.

It is estimated that as many as 7 out of 10 inpatients require intravenous catheterization. Poor practices in the insertion, maintenance and removal of intravenous catheters risk introducing pathogens directly to the bloodstream, which can lead to serious conditions such as sepsis, and difficult-to-treat complications in major organs like the brain and kidneys. Soft tissue infections at the insertion site of the intravenous catheter can also occur.

WHO will continue to work with countries to develop and implement best practices to reduce the occurrence of bloodstream infections in hospitals, and to ensure all patients receive safe and effective care.

https://bit.ly/4bdrzhD

## Global eye care initiative

WHO launched a new initiative, to assist countries in meeting the 2030 global eye care target of a 40% increase in the proportion of people with access to appropriate spectacles.

The “Specs 2030” initiative was launched during a meeting held in Geneva on 14 and 15 May, which brought together over 100 participants, including Member State representatives, United Nations agencies, academia and non-governmental organizations.

Uncorrected refractive error is the leading cause of vision impairment in child and adult populations, and more than 800 million people have a near-vision impairment (i.e. presbyopia) that could be corrected with a pair of reading spectacles.

https://bit.ly/3PXIBIe

**Looking ahead**

1 June. Seventy-seventh World Health Assembly Strategic Roundtable: Climate change and health. United Nations Palais des Nations, Geneva, Switzerland. https://bit.ly/3yrOh79

3–4 June. The 155th session of the Executive Board. World Health Organization, Geneva, Switzerland. https://bit.ly/4bEF4Hb

20–21 June. International dialogue on sustainable financing for noncommunicable diseases and mental health. Washington, United States of America. https://bit.ly/3QItRxk

